# A Single 10-Minute E-cigarette Vapor Exposure Reduces Tidal Volume and Minute Ventilation in Normoxia and Normobaric Hypoxia in Adult Rats

**DOI:** 10.7759/cureus.46735

**Published:** 2023-10-09

**Authors:** Jennifer A Stokes, Mila J Fisher

**Affiliations:** 1 Kinesiology, Southwestern University, Georgetown, USA; 2 Psychology, Southwestern University, Georgetown, USA

**Keywords:** breathing difficulty, vaping-associated lung injury, e-cigarette smoking, vaping, tidal volume, nicotine

## Abstract

The present study investigated the effects of a single 10-minute exposure to e-cigarette vapor on ventilation in adult male Long-Evans rats. Ventilation was recorded using awake, unrestrained whole-body plethysmography. Baseline recordings were taken the day before full-body exposure to either room air (n = 9; air control group) or e-cigarette vapor (n = 9; treatment group). Post-exposure recordings were taken immediately after the 10-minute room air or vapor exposure. As part of the ventilation protocol, in addition to recording the subject’s ventilation in room air, the subjects were also exposed to 10% oxygen (balanced with nitrogen) to assess the effects of e-cigarette vapor on an increased drive to breathe. Ventilation data were analyzed using a 2x2x2 mixed-model ANOVA measuring treatment (vape vs. air) x time (baseline vs. post-treatment) x condition (normoxia vs. hypoxia) for breathing frequency, tidal volume, and minute ventilation. Breathing frequency increased in both treatment groups (air and vape) with exposure to normobaric hypoxia (p < 0.001), with no effect of time (baseline vs. post-treatment) for either group. Tidal volume increased in both treatment groups (air and vape) with exposure to normobaric hypoxia (p < 0.001), and an effect of time (baseline vs. post-treatment) was observed (p = 0.010) for the vape group. Minute ventilation increased in both treatment groups (air and vape) with exposure to normobaric hypoxia (p < 0.001), and an effect of time (baseline vs. post-treatment) was observed (p < 0.001) for the vape group.

In conclusion, immediately following a single 10-minute e-cigarette vapor exposure, both tidal volume and minute ventilation were reduced during normoxia and normobaric hypoxia, indicating a decrease in ventilation after a single 10-minute e-cigarette vapor exposure. Furthermore, this exposure also blunted the physiological response to acute hypoxia exposure. Subjects in the vape group, while breathing more rapidly as expected, experienced shallower breathing than the air group during hypoxia. The findings in this study confirm that vaping could result in reduced lung function.

## Introduction

Vaping has been steadily increasing in popularity since its inception in 2003 [1]. In 2022, 2.55 million high schoolers and middle schoolers in the U.S. reported continuous vape usage, and of those who did, more than 27% vaped daily [2]. Additionally, in 2018, an estimated 5.2 million U.S. working adults used e-cigarettes, with 43.1% engaging in daily use [3]. As reviewed by Dinardo, despite vaping being touted as a safer alternative to smoking cigarettes, a steadily increasing body of research suggests that vaping leads to an abundance of adverse health effects associated with its usage [4]. As of early 2020, the CDC reported 2,807 cases of e-cigarette or vaping-associated lung injuries that required hospitalization [5]. Additionally, a 2019 study found that those who were hospitalized and diagnosed with an e-cigarette or vaping-associated lung injury showed symptoms of hypoxemia, fever, inflammatory response, and bilateral airspace opacification on imaging scans [6]. This growing body of research suggests that vaping is not a simple, harmless alternative to cigarette smoking.

E-cigarettes work by using an atomizer to heat an e-liquid mixture to create inhalable vapor [7]. The e-liquid commonly contains a solvent, such as propylene glycol or vegetable glycerin, a flavoring agent, and nicotine [7]. While both the common solvents and the various flavoring agents are considered safe for oral ingestion [8], there is no research suggesting that they are safe to be inhaled in an aerosolized form. Moreover, upon aerosolization, these agents have been found to release harmful chemicals such as acetone and formaldehyde [9]. A 2019 study acutely exposed human subjects to 25 e-cigarette puffs over five minutes and found deposits of propylene glycol and glycerin in the airway epithelium of human subjects, reaching all the way to the terminal bronchioles and alveoli [10]. The same study also found propylene glycol content in extracted serum, and this buildup is likely to cause respiratory harm [10]. Similarly, Kizhakke Puliyakote et al. (2021) found a decrease in mean alveolar ventilation in young human subjects after exposure to vaping, suggesting that vapor deposits may lead to airway restriction [11]. In acute animal exposure, a 15-minute e-cigarette exposure in anesthetized animals led to disruption of the alveolar-capillary membrane and impaired gas exchange, in addition to inflammation in rodent models [12]. Taken together, these studies suggest that even a single e-cigarette exposure can impair lung function and damage alveolar tissue.

In the present study, we aimed to investigate the effects of a single 10-minute e-cigarette vapor exposure on tidal volume, breathing frequency, and minute ventilation in awake rats. We administered either e-cigarette vapor or room air to adult male rats in a whole-body exposure chamber, and, immediately following this exposure, we recorded the subject’s ventilation using whole-body plethysmography. As part of the ventilation protocol, in addition to recording the subject’s ventilation in room air, the subjects were also exposed to 10% oxygen (balanced with nitrogen) to assess the effects of e-cigarette vapor on an increased drive to breathe. We hypothesized that this acute e-cigarette vapor exposure would negatively affect ventilatory function in both room air and normobaric hypoxia conditions due to airway restriction from the presence of the e-cigarette vapor chemicals, as indicated by previous research.

Portions of this article were previously presented as a meeting abstract and poster at the 2023 Texas Chapter of the American College of Sports Medicine Annual Meeting, February 23-24, 2023.

## Materials and methods

Subjects

For the experimental subjects, 18 adult male Long-Evans rats were used (weight range: 300-400 g). Rats were housed three per cage in hanging plastic cages on a metal cage rack, with aspen shavings used for bedding. Food and water were readily available at all times. Animal colony lighting was set at an automatic 12:12 hour reversed dark/light cycle, where the lights were turned off at 10:00 a.m., and humidity and temperature in the animal room were monitored daily. All ventilation recording and acclimation took place during the subject’s dark cycle under dim red light. Subjects were weighed every three days during acclimation and on the ventilation recording days. All animal protocols were approved by the Southwestern University Institutional Animal Care and Use Committee (IACUC), Georgetown, USA, under protocol Stokes_0721.

Air or e-cigarette vapor exposure procedure

Subjects were placed individually into a chamber either containing room air (n=9) or nicotine vapor (n=9; JUUL pod, 5% nicotine, Virginia tobacco flavor) for 10 minutes using a whole-body exposure chamber modeled after the OpenVape model developed by Frie et al. (2020) [13]. The vape exposure setup consisted of two polycarbonate cages with filter-top lids (26.7cm long, 16.5cm wide, and 15.9cm high), allowing for airflow while preventing vapor escape during the exposure sessions. Operating under negative pressure, two vacuum pumps drew two-second puffs from either a connected JUUL vaporizer equipped with a 5% Virginia tobacco pod or from the room air. The resulting e-cigarette vapor or room air was pumped into the respective exposure chambers (as described by Frie et al. [13]). Two subjects were run at a time, one exposed to air and one exposed to vaping, in individual exposure chambers. When turned on, the vape system ran a continuous program of two seconds of vape draw followed by a four-second break. This program ran for four minutes, after which the pumps were turned off and the subjects remained in the chambers for six additional minutes, totaling 10 minutes of vape or room air exposure. After exposure, the vape chamber was vented under a draft hood to allow for the safe escape of the vapor, and subjects were immediately placed in individual ventilation recording chambers.

Ventilation recording

**Figure 1:**
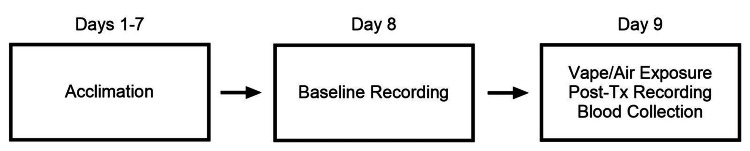
Experimental timeline Tx: treatment

Ventilation data were recorded via the Data Sciences International (DSI) Buxco (New Brighton, MN) whole-body plethysmography system with DSI rat plethysmography chambers. Whole-body plethysmography was performed on awake, unrestrained rats during the subject’s dark cycle under dim red light. Temperature and humidity were continuously monitored throughout the recording using a DSI-manufactured temperature-humidity probe, which was connected to the Buxco system. The gas (room air) flow rate was set to 3.0 L/min throughout the duration of the experiment, and the subjects were monitored continuously in the chamber. Each chamber was calibrated on recording days per the manufacturer's instructions. Subjects were acclimated in the ventilation chambers for seven consecutive days, 10 minutes each day, before baseline testing (Figure 1).

During acclimation, the subjects were placed in their assigned chambers and the system was turned on, with the lids sealed and room air flowing as described above, but recording was not enabled.

Baseline ventilation was recorded on the day before exposure (day eight; Figure 1) and followed the following paradigm: 10 minutes of acclimation (room air), 10 minutes of normoxia (room air), followed by 10 minutes of hypoxia (10% oxygen balanced with nitrogen; Linde Gas and Equipment Inc., Burr Ridge, IL). On the experimental day (day nine), the subjects were individually placed in the chambers immediately after 10 minutes of vaping or air exposure, as described above. Ventilation was recorded under the following conditions: 10 minutes of normoxia (room air), followed by 10 minutes of hypoxia (10% oxygen balanced with nitrogen; Linde Gas and Equipment Inc.). Subjects were placed back in their cages immediately after recording.

All ventilatory parameters were recorded and analyzed using the DSI FinePointe software (New Brighton, MN) (breathing frequency, tidal volume, and minute ventilation) using waveform calculations based on the Drorbaugh and Fenn equation [14]. Breathing frequency (breaths per minute) and tidal volume (the volume of air exchanged during a resting breath) were measured. Tidal volume was normalized to 100 grams of body weight. Minute ventilation (volume of breath per minute per 100 grams) was also calculated, as it is a product of the aforementioned parameters. In post-evaluation, in order to eliminate any data segments including sniffing or movement, the recordings were visually inspected for a smooth waveform representing a calm inhale and exhale, and 10x30-second clips were extracted from the exported data to use for statistical analysis.

Blood collection and processing

After all recordings had finished, subjects were euthanized with sodium pentobarbital (50 mg/kg; Covetrus Inc.; Portland, ME). Blood was collected directly from the heart via cardiac puncture, and then blood samples were left to sit at room temperature for 30-60 minutes without anticoagulant to allow for clotting. After coagulation, the blood samples were centrifuged at 2500 rpm for 15 min at 4°C, and the supernatant (serum) was extracted into a clean tube and stored at -80°C until use. Using a Cotinine ELISA kit (OriGene, Rockville, MD), blood cotinine levels were measured to confirm nicotine exposure. The manufacturer's instructions were followed.

Statistical analysis

All data analysis was performed using JASP, version 0.16.04 (University of Amsterdam, Amsterdam, Netherlands). Ventilation data was analyzed using a 2x2x2 mixed-model ANOVA measuring treatment (vape vs. air) x time (baseline vs. post-treatment) x condition (normoxia vs. hypoxia) for breathing frequency, tidal volume, and minute ventilation. Serum cotinine results were analyzed using a Student’s T-test.

## Results

Ventilation was recorded in normoxia (room air) and normobaric hypoxia (10% oxygen) both before (baseline) and immediately after a single 10-minute air or vapor exposure (post-tx). Breathing frequency and tidal volume were measured. Tidal volume was normalized to 100 grams of body weight. Minute ventilation (volume of breath per minute per 100 grams) was also calculated, as it is a product of the aforementioned parameters.

**Figure 2:**
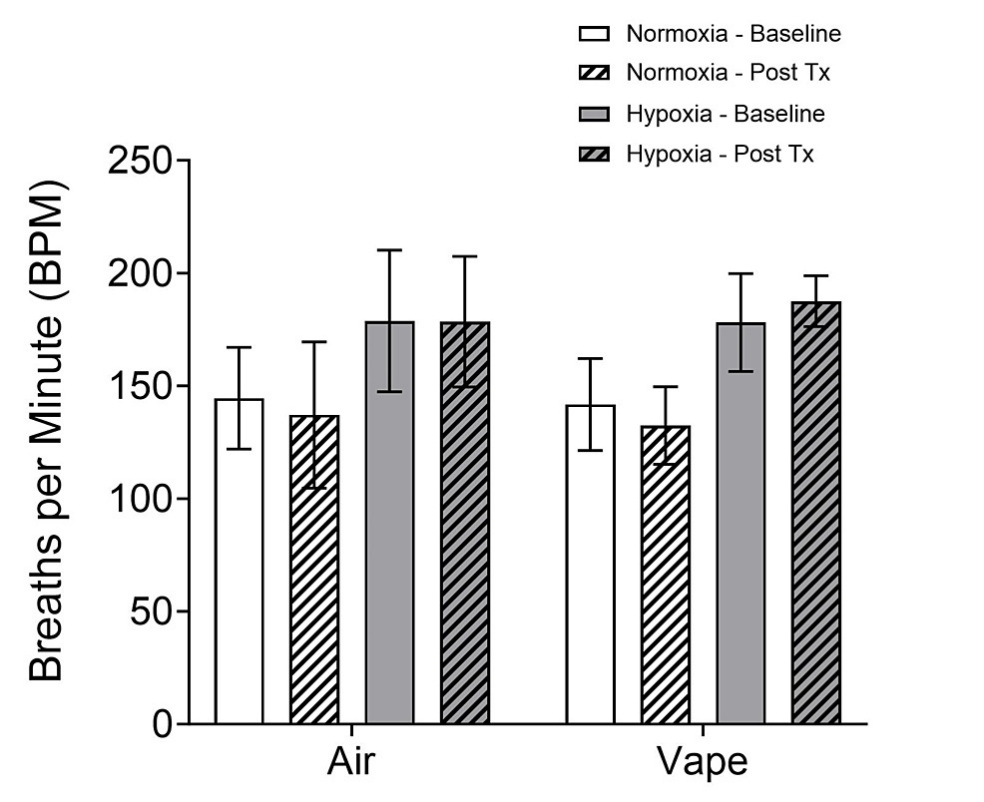
Breathing frequency before (baseline) and after treatment Breathing frequency increased in both treatment groups (air and vape) with exposure to normobaric hypoxia (p < 0.001), with no effect of time (baseline vs. post-treatment) for either group. Tx: treatment

Breathing frequency (Figure 2) increased in both treatment groups (air and vape) with exposure to normobaric hypoxia (p < 0.001) with no effect of time (baseline vs. post-treatment) for either group (2x2x2 mixed-model ANOVA; treatment x time x condition).

The subjects increased their rate of breathing during hypoxia, regardless of treatment.

**Figure 3:**
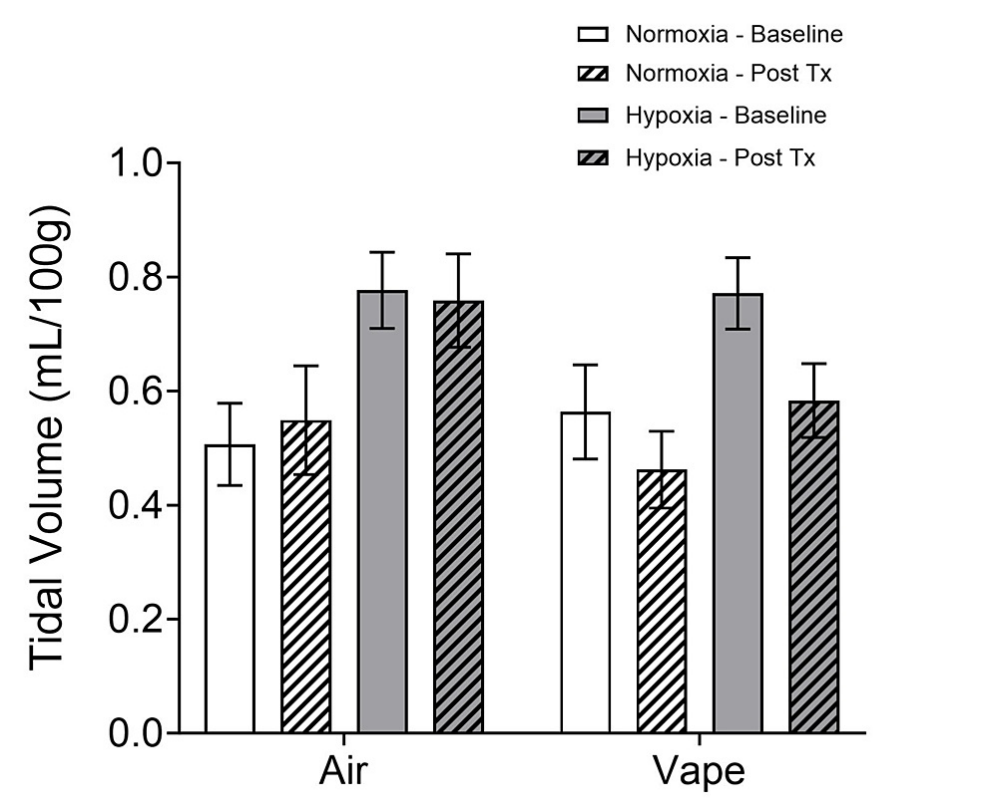
Tidal volume before (baseline) and after treatment. Tidal volume increased in both treatment groups (air and vape) with exposure to normobaric hypoxia (p < 0.001) and an effect of time (baseline vs. post-treatment) was observed (p = 0.010) for the vape group. Tx: treatment

Tidal volume (Figure 3) increased in both treatment groups (air and vape) with exposure to normobaric hypoxia (p < 0.001), and an effect of time (baseline vs. post-treatment) was observed (p = 0.010) for the vape group (2x2x2 mixed-model ANOVA; treatment x time x condition).

Following treatment, the air subjects increased their breath volume under hypoxic conditions, but the vape subjects did not.

**Figure 4:**
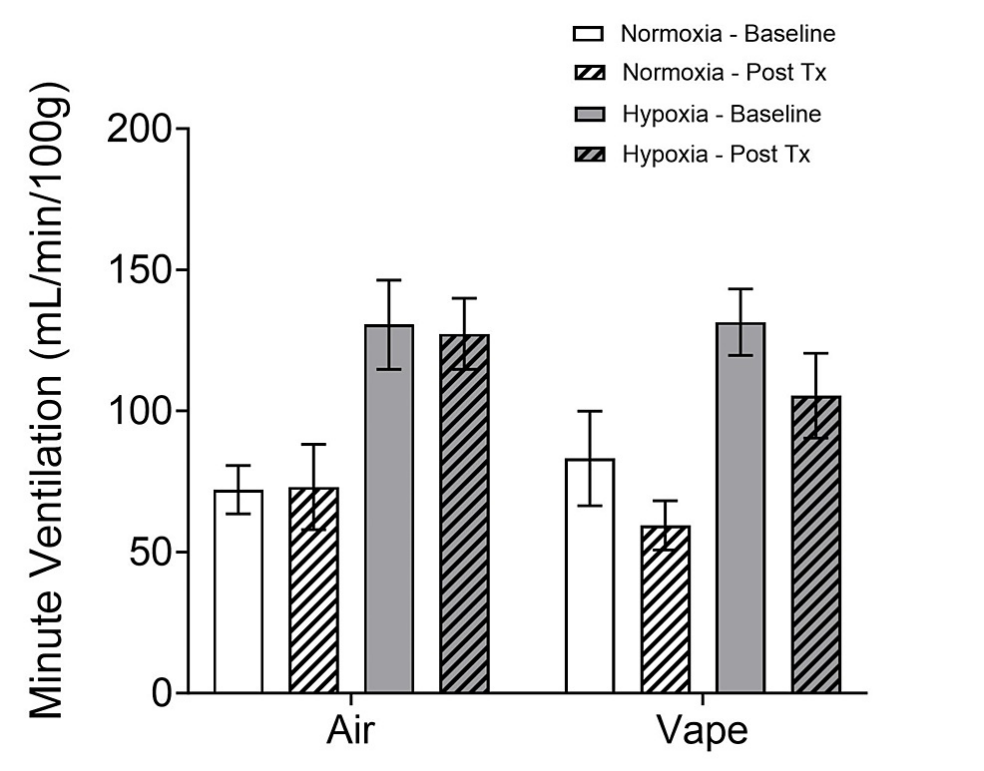
Minute ventilation before (baseline) and after treatment Minute ventilation increased in both treatment groups (air and vape) with exposure to normobaric hypoxia (p < 0.001), and an effect of time (baseline vs. post-treatment) was observed (p < 0.001) for the vape group. Tx: treatment

Minute ventilation (Figure 4) increased in both treatment groups (air and vape) with exposure to normobaric hypoxia (p < 0.001), and an effect of time (baseline vs. post-treatment) was observed (p < 0.001) for the vape group (2x2x2 mixed-model ANOVA; treatment x time x condition).

Following treatment, the air-exposed subjects increased their minute ventilation under hypoxic conditions, but the vape-exposed subjects did not. Since minute ventilation is a function of frequency and tidal volume, the decrease in tidal volume (Figure 3) without a corresponding change in frequency (Figure 1) results in a decrease in minute ventilation after vape exposure.

To confirm the experimental e-cigarette vapor exposure condition, an assay that detects cotinine (a nicotine metabolite) was performed on serum samples. Cotinine was present in the serum of the vapor-exposed group (89.16 ng/mL ± 31.16 ng/mL) with negligible levels in the air control group (0.054 ng/mL ± 0.051 ng/mL), confirming nicotine vapor exposure in the vapor-exposed group only (p < 0.001, Student’s t-test).

## Discussion

In this study, we assessed the effects of a single 10-minute exposure to e-cigarette vapor on ventilation parameters in adult male rats. Subjects were exposed to either room air or vape via whole-body exposure, and both baseline and post-treatment ventilation parameters were measured using awake, unrestrained whole-body plethysmography. During ventilation recording, subjects were exposed to both normoxia (room air) and normobaric hypoxia (10% oxygen) to assess the effects of e-cigarette vapor on an increased drive to breathe. We found that all subjects, regardless of the treatment group, increased breathing frequency during normobaric hypoxia, as expected [15]. Given the decrease in environmental oxygen to 10%, an increase in both frequency and tidal volume is expected due to stimulation of peripheral (carotid body) chemoreceptors, which are sensitive to changes in arterial oxygen content. Post-treatment, this same phenomenon was observed for the air group, indicating that the room air exposure did not alter the physiological response to acute hypoxia exposure. However, immediately following a single e-cigarette vapor exposure, both tidal volume and minute ventilation were reduced during normoxia and normobaric hypoxia, indicating a decrease in ventilation in both normoxic conditions and a blunting of a physiological response to acute hypoxia exposure. During the hypoxia challenge, subjects in the vape group, while breathing more rapidly as expected, experienced shallower breathing than the air group.

The decrease in tidal volume in both normoxia and hypoxia after vape exposure could be explained by protective bronchoconstriction. Khosravi et al. (2018) found that a single puff of nicotine vapor induced around two minutes of acute bronchoconstriction in adult guinea pigs, mediated by cholinergic pathways; air controls did not experience the same bronchoconstriction [16]. Additionally, in the same study, when vapor not containing nicotine was administered, similar effects of bronchoconstriction occurred [16], suggesting that the vehicle vapor alone has negative effects on ventilation. Given that the subjects in the present study were exposed to 10 minutes of continuous e-cigarette vapor rather than only a single puff, the present effects of decreased tidal volume during hypoxia could be explained by the onset of a protective bronchoconstriction reflex, triggered by the irritating presence of e-cigarette vapor in the lungs. However, since the present study did not test the effects of a vehicle-only control or individual chemical controls (e.g., propylene glycol), the decrease in tidal volume and minute ventilation observed here cannot be attributed to a single vapor component. Initiation of a tissue inflammatory response may also play a role, as noted in a study by Lim and Kim (2014) in adolescent mice, which found an effect of bronchoconstriction attributed to the infiltration of inflammatory cells into the airway [17]. However, this study exposed mice to e-cigarette liquid directly from the cartridge, administered intratracheally [17]. The present study did find similar effects to these studies in guinea pigs and mice, even though our adult rat subjects were exposed to e-cigarette vapor using full-body exposure.

In the future, we would like to expand upon these results to include an assessment of the duration of the reduction in tidal volume following a single 10-minute vapor exposure as well. On the molecular biology side, with serum and lung tissue samples, we would also like to investigate the molecular changes that may occur in the lungs by measuring inflammatory biomarkers and tissue damage, as there is research suggesting that vaping could cause alveolar dysfunction, disruption of the alveolar-capillary barrier, and increased pulmonary inflammation [12].

Limitations

The full-body exposure in this experiment is limited in its external validity because vaping in humans is carried out via direct inhalation. However, it does provide a model for intense second-hand exposure, suggesting that second-hand vapor exposure may also affect lung function. However, given that the present study yielded significant results with whole-body exposure, it is reasonable to conclude that direct inhalation of e-cigarette vapor could cause exacerbated effects on ventilation parameters. And, as noted above in the context of other work, without a vehicle-only group, the effects observed cannot be attributed to a single vapor component. This experiment also only included male subjects because female hormonal cyclicity has effects on breathing parameters [18]. An additional difference between the present study and the body of vape research with rodent models is the strain of rats used. The present study used male Long-Evans rats; there are not any studies on acute vape exposure in Long-Evans rats, a strain commonly used in behavioral studies.

## Conclusions

In conclusion, vaping has been shown to have numerous negative effects, specifically on lung structure and function. To add to the present body of vape research, this study found that an acute e-cigarette exposure of 10 minutes induces functional effects of reduced tidal volume and minute ventilation in adult male Long-Evans rats in both normoxic and hypoxic environments.
